# Bridging the Gap: The Transition Toward University-Level Midwifery Education and Autonomous Practice in Ukraine

**DOI:** 10.7759/cureus.111602

**Published:** 2026-06-27

**Authors:** Igor Lakhno, Victoria Romaieva, Svitlana Pak, Iryna Sykal

**Affiliations:** 1 Obstetrics and Gynecology, Kharkiv National Medical University, Kharkiv, UKR

**Keywords:** bologna process, eu directive 2005/36/ec, ivan lazarevich, maternal health, midwifery, physiological birth, professional autonomy, simulation-based education sbe, srhr competencies, ukraine

## Abstract

Midwifery in Ukraine is currently undergoing a transformative shift from deep-seated traditional roots toward professional autonomy and alignment with international standards. Historically, care transitioned from village midwives, known as babas or spovytukhas, to formalized training in the 18th century, and scientific professionalization led by figures such as Ivan Lazarevich, who championed a physiological approach to childbirth. However, the Soviet period introduced a physician-led, medicalized model that often integrated midwifery with nursing at a sub-degree level.

Currently, the profession faces acute modern pressures caused by the 2022 full-scale invasion, which has disrupted services, forced births into shelters, and contributed to a rise in pregnancy complications. Systemic challenges include significant workforce shortages and curriculum gaps; a 2023-2024 United Nations Population Fund (UNFPA) assessment found that Ukrainian curricula cover a good percentage of key sexual and reproductive health and rights (SRHR) competencies, but not all.

To address these issues, Ukraine is leveraging the Bologna Process and European Union (EU) accession aspirations to modernize education toward Bachelor of Science (BSc), Master of Science (MSc), and doctoral (PhD) levels. International collaborations, such as the 'Midwifery Bridges' project with Sweden, aim to adopt autonomous care models. Furthermore, simulation-based education has emerged as a critical technological tool for safely developing clinical dexterity and emergency preparedness amid wartime disruptions. Ultimately, these reforms seek to establish midwives as independent, responsible birth attendants providing woman-centered, evidence-based care.

## Introduction and background

Legacy of soviet integration

Midwifery in Ukraine is currently undergoing a significant transition, moving from a medicalized, physician-led model toward professional autonomy and academic advancement aligned with international standards. To understand this shift, it is essential to consider the structural legacy of the Soviet healthcare system, in which midwifery was integrated into nursing as a sub-degree specialty. In the Ukrainian context, 'sub-degree level' refers to vocational or associate-level training, typically 29-month specialized tracks, rather than the four-year university bachelor’s degrees common in the European Union (EU). Under this model, midwives functioned as technical assistants within a physician-led hierarchy rather than as independent practitioners [1]. Today, the regulatory landscape is governed by the Ukrainian Council of Nursing and Midwifery (part of the Ukrainian Medical Council), which manages registration and certification. Simultaneously, the Ukrainian Midwives' Union advocates for professional development and the legal expansion of the midwifery scope of practice toward autonomous care. However, despite these efforts, midwives often remain legally and structurally linked to nursing, lacking full autonomy in routine obstetric care [2].

Scope of the review

The scope of this review provides a targeted analysis of the Ukrainian midwifery landscape across three distinct temporal and thematic boundaries. (1) Historical development (18th century to 1991): tracing the transition from traditional village practitioners (babas) to the formal scientific schools established by Ivan Lazarevich. (2) Educational reform (2005 to present): evaluating the impact of the Bologna Process and the shift toward establishing university-level Bachelor of Science (BSc), Master of Science (MSc), and doctoral (PhD) programs aligned with EU Directive 2005/36/EC. (3) Technological prospects and resilience (post-2022): analyzing the integration of simulation-based education (SBE) as a strategy to maintain clinical training quality amidst war-related disruptions. While the primary focus is on Ukraine, this review situates national progress within the broader Eastern Europe and Central Asia (EECA) region to highlight comparative gaps in competency coverage. 

Wartime resilience and modern pressures

The purpose of this synthesis is to evaluate how Ukraine is leveraging international standards to build professional resilience. The 2022 full-scale invasion has forced an immediate adaptation of midwifery practice, as births are frequently conducted in shelters and basements under high-stress conditions. This conflict has led to a 12% rise in pregnancy complications, such as premature births and stress-induced C-sections, while simultaneously causing a 30% decline in the midwifery and nursing workforce due to displacement and injury [3]. This review examines how these acute pressures have accelerated the adoption of technology, such as virtual reality and high-fidelity simulation, to ensure midwives can function as sole responsible attendants in emergency settings.

Justification for the review

Despite these profound shifts, no comprehensive synthesis of the Ukrainian midwifery transition currently exists in English-language literature. This review addresses that gap by documenting how the intersection of historical legacy, EU integration aspirations, and wartime necessity is forging a new model of autonomous, woman-centered midwifery in Ukraine.

## Review

Methodology

Algorithm for Selection of Sources

This review utilizes a dual-algorithm approach to identify and select evidence, ensuring the content is grounded in both historical context and modern evidence-based practice. The search strategy targeted primary databases (PubMed, Google Scholar, and European Journal of Midwifery) and institutional repositories (United Nations Population Fund (UNFPA), WHO, and International Confederation of Midwives (ICM)) for literature published from 2005 onwards, with a specific focus on post-2022 wartime impacts.

**Figure 1:**
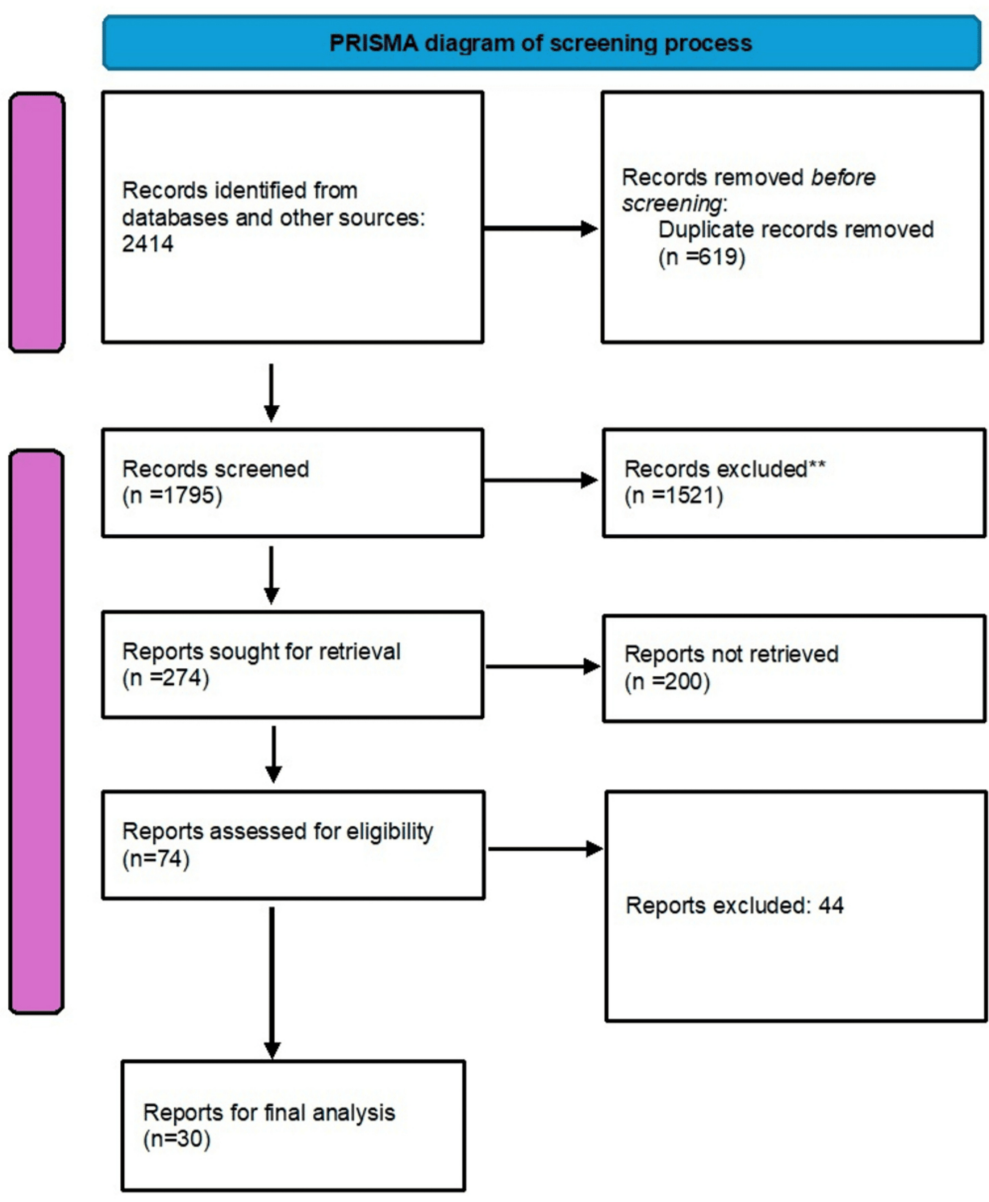
Flowchart illustrating the selection process for studies included in this review PRISMA: Preferred Reporting Items for Systematic Reviews and Meta-Analyses [4]

To ensure a transparent and reproducible selection process, this study adheres to the Preferred Reporting Items for Systematic Reviews and Meta-Analyses (PRISMA) guidelines [4]. The stages of paper selection, including identification, screening, and eligibility, are detailed in the PRISMA diagram of the screening process (Figure 1). While this review utilizes the PRISMA framework to ensure a transparent and reproducible selection process, it is formally classified as a narrative review. The study is a narrative review using a systematic search strategy.

The methodological distinction is defined by how the evidence is synthesized. The systematic selection process employs the PRISMA 2020 framework as a rigorous tool to identify, screen, and select evidence. This structured approach ensures the study is grounded in modern evidence-based practice and international standards. Despite the systematic selection of those 30 sources, the final synthesis is presented as a narrative review. The PRISMA framework is employed here to ensure methodological rigor in identifying the review's core pillars: historical development, educational standards, and technological prospects.

This algorithm defines the criteria for identifying and selecting the evidence used to construct the review. The search strategy and identification involved a search on primary databases such as PubMed, Google Scholar, and the European Journal of Midwifery for peer-reviewed studies on "midwifery in Ukraine," "Bologna Process in health education," “midwifery education," and "simulation-based education." 'Grey literature' was identified from key international and national bodies, specifically the United Nations Population Fund Eastern Europe and Central Asia (UNFPA EECA), the WHO, the ICM, and the Ukrainian Midwives’ Union. The temporal scope prioritized sources from 2005 onwards (alignment with the Bologna Process) and post-2022 (impact of the full-scale invasion on maternal health).

Inclusion and Exclusion Criteria

Sources that specifically address the Ukrainian healthcare system or the broader Eastern Europe and Central Asia (EECA) region, inclusive of Ukraine; that cover at least one core pillar: historical development (e.g., babas/spovytukhas), educational standards (e.g., EU Directive 2005/36/EC [2]), or technological prospects (e.g., simulation-based education); and that are English and Ukrainian language publications featuring both international standards and local professional nuances were included in the study. General nursing studies that do not specifically address midwifery competencies or autonomy, or outdated medical models that do not reflect the current shift toward woman-centered, physiological birth, were excluded. A total of 30 studies were selected for their comprehensive view of the professionalization of midwifery, including historical roots, current wartime resilience, and the transition to simulation-supported, university-level education. Table 1 categorizes the studies identified through the PRISMA process.

**Table 1:** Key findings of the included reports EU: European Union, ICM: International Confederation of Midwives, VR: Virtual reality, EECA: Eastern Europe and Central Asia, OSCEs: Objective structured clinical examinations, UNFPA: United Nations Population Fund, EUA: European University Association, BSc: Bachelor of Science, MSc: Master of Science, PhD: Doctoral program, SBE: Simulation-based education

Author/source, year, and reference no.	Key findings/contribution to review	Category
Boryak (2003) [1]	Describes traditional Ukrainian midwives (babas or spovytukhas) and their role in rural culture using herbal knowledge, folklore, and rituals.	Historical and cultural
Drandic et al. (2026)[2]	Argues that updating EU directives presents a 'generational opportunity' to align professional qualifications with modern standards and autonomous practice.	Historical and cultural
Lakhno (2022) [3]	Documents a 12% rise in pregnancy complications near the frontlines in Kharkiv and the reality of births occurring in shelters.	Education standards (EU/ICM) and wartime Impact
Page (2021) [4]	Methodological aspects of the review.	Methodology of the review
Sokol (2009) [5]	Notes that traditional midwives continued to attend the majority of rural births well into the 1920s and 1930s, even as formal training began to expand.	Historical and cultural
Vinnitskiy & Shulga (2021) [6]	Highlights Ivan Lazarevich as a foundational figure who established a scientific midwifery school in Kharkiv and pioneered the physiological approach to childbirth.	Historical and cultural
Cummins & Gilkison (2023) [7]	Analyzes the historical integration of midwifery with nursing and the broader medicalized healthcare system.	Historical and cultural
Leoniuk et al. (2025) [8]	Explores midwives' experiences caring for refugees and the role of international cross-border support (e.g., Polish midwives).	Education standards (EU/ICM) and Wartime Impact
Nenko et al. (2024) [9]	Reports a 30% decline in nursing and midwifery personnel between 2015 and 2022 due to low pay, migration, and poor conditions.	Education standards (EU/ICM) and Wartime Impact
Lakhno (2025) [10]	Analyzes the long-term impact of wartime stress on perinatal outcomes and the need for mental health support.	Education standards (EU/ICM) and Wartime Impact
Brigante et al. (2025) [11]	Outlines systemic barriers to autonomy, including healthcare funding challenges and a reliance on out-of-pocket payments.	Education standards (EU/ICM) and Wartime Impact
UNFPA EECA (2022) [12]	Evaluates the regional midwifery workforce and supports the development of policy frameworks for emergency response.	Education standards (EU/ICM) and Wartime Impact
Patterson et al. (2019) [13]	Surveys alumni to reveal a traditionally physician-led, medicalized model of childbirth at the secondary or associate education levels.	Education standards (EU/ICM) and Wartime Impact
EUA (2026) [14]	Provides the framework for the Bologna Process, facilitating the shift toward a three-cycle higher education system (BSc, MSc, PhD).	Education standards (EU/ICM) and Wartime Impact
Graf et al. (2020) [15]	Discusses the 'academization' of midwifery and the implementation of doctoral (PhD) research programs.	Bologna Process and academic degrees
Mivšek et al. (2016) [16]	Details the availability and necessity of MSc programs for advanced clinical practice, leadership, and research roles in midwifery.	Bologna Process and academic degrees
Swedish Institute (2025-2026) [17]	Outlines the 'Midwifery Bridges' project with Sweden to adopt autonomous midwifery models in Ukraine.	Bologna Process and academic degrees
Yahya et al. (2024) [18]	Provides a systematic review supporting immersive simulation (SBE and virtual reality (VR)) as a safe way to build dexterity and preparedness.	SBE
O'Connell (2025) [19]	Analyzes the system of midwifery education in Europe, compares regional progress, noting that while all EU countries offer bachelor’s degrees, only half of the EECA countries do so.	Policy and regional assessments
UNFPA EECA (2024) [20]	Specifically identifies curriculum gaps in Ukraine, noting that HIV/STI inclusion is as low as 4% in some metrics.	Policy and regional assessments
WHO (2019) [21]	Establishes the framework for action on quality midwifery education to achieve Universal Health Coverage 2030.	Policy and regional assessments
Adnani et al. (2022) [22]	Highlights barriers to preparing midwifery students for obtaining adequate midwifery knowledge and skills during clinical placement	Policy and regional assessments
Akmese et al. (2024) [23]	Studies online communication skills programs as a practical pathway for adaptable training during crises.	SBE
Scamell & Hanley (2017) [24]	Examines innovation in preregistration education, including web-based interactive storytelling for learning.	SBE
Hernon et al. (2023) [25]	Reviews the use of educational technology (e.g., Peyton’s four-step approach) for teaching psychomotor skills.	SBE
Urbanová et al. (2018) [26]	Details the creation and benefits of virtual patients in midwifery education for safe, repeatable practice.	SBE
Cullen et al. (2003) [27]	Validates the use of interprofessional objective structured clinical examinations (OSCEs) for assessing teamwork and decision-making among medical and midwifery students.	SBE
Maskálová et al. (2018) [28]	Analyzes lecturers' experiences with simulation training and the importance of faculty development.	SBE
McTague & Smith (2023) [29]	Provides a scoping review protocol for using simulation in advanced midwifery practitioner education.	SBE
Cooper et al. (2012) [30]	Confirms the educational and clinical impact of simulation-based learning on skills, confidence, and teamwork.	SBE
Elendu et al. (2024) [31]	Reinforces the broad impact of simulation-based training in medical education, supporting modern evidence-based practice.	SBE

Study Identification Process

Records were identified through databases using the keywords "midwifery Ukraine," "maternal health war impact," and "Bologna process healthcare." Additional records were identified through other sources, such as the reports from UNFPA (2022-2024), Swedish Institute (Midwifery Bridges project), and WHO frameworks. These studies were then screened to remove duplicates, and titles/abstracts were screened for relevance to identify the shift from physician-led to autonomous midwifery. Full-text assessment was carried out to evaluate sources for data on sexual and reproductive health and rights (SRHR) competencies (noting the identified 49% to 66% coverage gap) and alignment with ICM global standards [20]. An educational-level analysis was conducted by categorizing the identified sources according to their specific contributions to the prospects of BSc, MSc, and PhD programs. Figure 1 illustrates the process used to identify the selected studies.

Results

Historical Development of Ukrainian Midwifery

Midwifery in Ukraine has deep historical roots, originally centered on traditional village midwives known as babas or spovytukhas. These women, often widows with extensive practical experience, handled the majority of births in rural areas using a combination of herbal knowledge, folklore, and spiritual rituals. These midwives attended most births, performing practical tasks (e.g., cutting the umbilical cord, swaddling) alongside symbolic and spiritual elements. Terms for midwives varied regionally but emphasized their roles in delivery and care [2].

Formal medicalized midwifery began to emerge in the late 18th century with the establishment of obstetrics schools in cities like Chernivtsi (1783) and Kyiv (1803). While the Zemstvo reforms of the 1870s expanded training, traditional midwives continued to attend most rural births well into the 1920s and 1930s. The Soviet era further professionalized the field through maternity hospitals, though care remained largely physician-led rather than autonomous [5].

Ivan Lazarevich (1829-1902) is widely regarded in historical medical literature as a foundational figure in Ukrainian midwifery. Lazarevich was a professor at Kharkiv University, where he established a highly influential midwifery school. This school was instrumental in the transition from traditional folk practice to scientific professionalization. He was a pioneer of the physiological approach to childbirth. This historical focus aligns with the modern goals mentioned in your source to move away from a 'medical-model focus' and back toward 'physiological birth and woman-centered care.' He is famous for inventing the Lazarevich forceps, which were designed to be parallel and non-crossing to reduce trauma to the mother and infant during delivery. His midwifery school was transformed into the Kharkiv Female Medical Institute later [6].

While the provided source focuses on the current shift toward BSc, MSc, and PhD levels and alignment with EU Directive 2005/36/EC [2], Lazarevich’s 19th-century work in Kharkiv [6] laid the groundwork for the academic midwifery education that Ukraine is currently working to modernize. His emphasis on specialized training for midwives as distinct from general medicine mirrors the current effort to establish midwifery as an autonomous profession. The study by Cummins & Gilkison showed the historical integration of midwifery with nursing [7]. Soviet-period developments further professionalized care through maternity hospitals and collective farm facilities, though access and quality varied. Post-independence, midwifery has been part of the broader healthcare system, often linked to nursing [5].

Current State of Midwifery in Ukraine

Since the 2022 invasion, the maternal health system has faced severe disruption, with births occurring in shelters and a 12% rise in complications due to stress and trauma. Workforce shortages have become acute, with nursing and midwifery personnel numbers dropping by approximately 30% between 2015 and 2022 [3]. Today, regulation is managed by the Ukrainian Council of Nursing and Midwifery and the Ukrainian Midwives' Union, which advocate for professionalization despite funding challenges and systemic barriers to autonomy.

Regulation and Midwifery Education Today

Midwives in Ukraine are regulated through bodies like the Ukrainian Council of Nursing and Midwifery (part of the Ukrainian Medical Council), which handles registration, certification, and efforts toward EU integration. There is also the Ukrainian Midwives' Union, which focuses on professional development, education standards, and support for women's health. Education typically involves secondary education plus specialized training (often two to three years), but it is frequently below the bachelor's level and integrated with nursing, unlike many EU countries that emphasize independent midwifery degrees. A 2023-2024 UNFPA assessment of midwifery education in EECA (including Ukraine) found that curricula cover only about 49% to 66% of key SRHR competencies on average, with particularly low inclusion in areas like HIV/sexually transmitted infections (STIs) (e.g., ~4% in some reports), infertility, and comprehensive sexuality education [20]. Gaps exist in hands-on clinical training, resources, and alignment with the ICM global standards [7]. There are ongoing initiatives, such as partnerships (e.g., with Sweden), to modernize education toward EU standards.

Current Challenges

Midwifery and the broader maternal health system in Ukraine confront multiple, interconnected issues. Especially the impact of war and conflict. Since 2022, the full-scale Russian invasion has severely disrupted services. Births occur in shelters, basements, or under shelling; maternity hospitals have been attacked. Stress has increased complications (e.g., a ~12% rise in one hospital, with more C-sections and premature births) [3]. Midwives and healthcare workers operate with limited electricity, water, and supplies, and face personal risk. Many have been displaced, joined the military, emigrated, or been killed/injured. Pregnant women face heightened risks from malnutrition, poor sanitation, and trauma. International aid (kits and training) and cross-border support (e.g., Polish midwives aiding refugees) have helped, but needs remain acute [8].

Like nursing, midwifery has seen declines (nurses dropped ~30% from 2015 to 2022 due to low pay, heavy workloads, and poor conditions). Similar pressures affect midwives. Exhaustion, emotional strain, and migration exacerbate shortages, especially in rural or frontline areas [9].

Healthcare funding challenges, reliance on out-of-pocket payments in some cases, and incomplete reforms that limit access are some of the systemic issues around resources. Midwifery is not always fully autonomous or distinct from nursing. The SRHR integration lags affect comprehensive care. Smaller facilities struggle with sustainability [5]. In the broader context, demographic pressures (low birth rates, aging populations, and war-related losses), refugee experiences (e.g., Ukrainian women in neighboring countries noting differences in care models), and the need for better mental health support and continuity of care add complexity [10].

Professional organizations are pushing for stronger regulation, registration, education upgrades, and international alignment. International partners (WHO, UNFPA, ICM) support workforce strengthening, policy development, and emergency response. Midwives continue to play a vital role in resilience, with stories of dedication amid adversity highlighting their importance [11]. Progress depends on ending the conflict, sustained investment in training and retention, and policy reforms to elevate midwifery as an autonomous profession. For the latest developments, consulting sources like the Ukrainian Midwives' Union or the WHO/Europe reports is recommended. Midwifery education in Ukraine is undergoing a gradual shift toward alignment with European and international standards, driven by EU integration aspirations, professional advocacy, and post-Soviet reforms, though significant gaps persist amid wartime challenges [12]. 

Reform Drivers and International Alignment

Following the Bologna Process (2005), Ukraine is shifting toward a three-cycle system (BSc, MSc, PhD) [14]. However, current curricula cover only 49% to 66% of essential SRHR competencies, with a significant lack of training in HIV/STIs and safe abortion care. Modernization efforts now emphasize SBE, which uses low- and high-fidelity trainers to safely build clinical dexterity and emergency preparedness in high-stakes environments. Projects like 'Midwifery Bridges' with Sweden are pivotal in transitioning the Ukrainian workforce toward an autonomous midwifery model, where midwives serve as the primary responsible attendants for physiological births and family planning. Traditionally integrated with nursing or medical training, midwifery in Ukraine (and much of the post-Soviet space) emphasized a more medicalized model of childbirth. The study by Patterson et al. showed that education was often at a secondary or associate level rather than a full bachelor's degree, with curricula reflecting a physician-led approach rather than autonomous midwifery practice [13].

Ukraine joined the Bologna Process in 2005, initiating a broader shift in higher education toward a three-cycle system (Bachelor's, Master's, and PhD); European Credit Transfer and Accumulation System (ECTS) credits; and quality assurance aligned with the European Higher Education Area (EHEA). This provided a framework for modernizing health professions education, though implementation in midwifery has been slower and inconsistent compared to general higher education reforms [14]. There is a strong and growing prospect for midwifery education at BSc, MSc, and PhD levels, particularly in Europe and as part of Ukraine’s ongoing higher education reforms aligned with the Bologna Process [14] and EU integration.

The BSc level is an established standard in Europe. Direct-entry BSc (or equivalent) midwifery programs (typically three to four years) are the norm across the EU, meeting EU directive 2005/36/EC requirements [2] and aligning with the ICM global standards. These programs combine theory, simulation, and extensive clinical practice. In Ukraine, midwifery education has historically been below the bachelor's level (often integrated with nursing at the secondary or associate level). However, reforms under the Bologna Process and EU accession aspirations are shifting toward degree-level qualifications [14]. Just over half of EECA countries (including some progress in Ukraine) now offer the equivalent of bachelor’s programs, with ongoing efforts to elevate standards. Many Ukrainian institutions are modernizing curricula, and international partnerships (e.g., with Sweden) support this transition. A BSc provides the foundation for autonomous, competent practice.

The MSc level is well-established in Europe. Numerous MSc programs exist for qualified midwives focusing on advanced clinical practice, leadership, education, research, or specialized care (e.g., enhanced midwifery care). These prepare midwives for advanced roles beyond entry-level practice. In Ukraine/EECA, postgraduate qualifications in midwifery are limited (only a few EECA countries offer them), but demand is rising with workforce needs and professionalization efforts. Reforms in higher education (updated standards, university autonomy) create pathways for new master’s programs. Prospects are promising. Master’s programs address gaps in management, teaching, and research that a bachelor’s alone cannot fully meet. They are key to career progression and faculty development.

The PhD level is available and growing in Europe. The PhD programs in midwifery (or nursing/midwifery) exist at universities in the UK, Ireland, and other countries. The study by Graf et al. emphasized the need for midwifery education academization and the prospect for PhD training courses [15]. These focus on original research in areas like maternal health, clinical outcomes, education, or policy. Entry typically requires a strong master’s and relevant experience [16]. In Ukraine, doctoral-level education follows the three-cycle Bologna structure (Bachelor-Master-PhD), but specific midwifery PhDs are not yet widespread. Broader higher education reforms and integration into the EHEA facilitate the development of research programs. Prospects are emerging but viable. Doctoral degrees are essential for building midwifery research capacity, academic leadership, and evidence-based practice. International collaborations and ICM/WHO advocacy support this trajectory, especially for countries like Ukraine aiming for EU alignment.

Overall Outlook and Drivers

Both the ICM and WHO support global standards that emphasize rigorous, competency-based education at higher levels to strengthen the profession. Simulation, clinical experience, and research integration are encouraged across all levels. Integration with the EU, Bologna reforms, and projects like 'Midwifery Bridges' are accelerating progress. Challenges include resources, war impacts, and faculty shortages, but the direction is toward full degree-level (and postgraduate) pathways. Especially as higher-level education improves clinical outcomes, professional autonomy, leadership, and retention. It also supports teaching the next generation of midwives. New initiatives, such as the 'Midwifery Bridges' project with Sweden (2025-2026), aim to adopt Swedish autonomous models and update clinical training. There is also a growing emphasis on SBE to safely build clinical skills and emergency preparedness [17]. Yahya demonstrated the use of medical simulation in the process of midwife education [18].

Across Europe, midwifery education has evolved under the EU Directive 2005/36/EC (on recognition of professional qualifications), which sets minimum standards (e.g., three years/4600 hours for direct-entry or 18 months post-nursing) [2]. However, the directive's core education provisions have seen limited updates since the 1980s, prompting ongoing calls for alignment with ICM Global Standards for Education and Essential Competencies. Many EU countries have shifted to university-level (bachelor’s) direct-entry programs, emphasizing autonomy, evidence-based practice, and continuity of care. The Bologna Process has facilitated mobility and harmonization, though variations remain (e.g., dual university-practical models in places like Germany) [14]. Efforts continue to update the EU Directive for better integration of modern evidence, technology, and SRHR. The study by O'Connell revealed the peculiarities and trends in midwifery education [19]. The EECA countries show diversity (e.g., Türkiye, Serbia) and have higher SRHR coverage and degree-level qualifications, while others lag. All EU countries offer bachelor's or higher, compared to only about half in EECA [20,21].

Reforms in higher education and healthcare are tied to alignment with EU integration, including professional qualification recognition. International projects support modernization through training, internships, policy dialogue, and adoption of autonomous midwifery models. Adnani et al. revealed the issues of clinical education in midwifery in Indonesia [22]. These problems are internationally recognized and relevant to the Ukrainian educational environment. 

Professional and international advocacy via partnerships with UNFPA, WHO, ICM, and the Ukrainian Midwives' Union push for ICM-aligned curricula, better regulation, and elevated status. Broader higher education reforms through updates of degree standards, university autonomy, and qualifications frameworks (e.g., 2024-2025 resolutions [20]) facilitate integration into the EHEA. While the ongoing conflict has accelerated the need for adaptable, high-quality training while disrupting implementation, it underscores midwives' critical role.

Discussion

Challenges and Barriers

Moving from nursing-integrated, sub-degree programs to independent bachelor's remains a hurdle. Under modern autonomous models, midwives are expected to independently manage normal pregnancies. Their role in antenatal care involves monitoring the health of the mother and fetus, providing education, and referring patients to medical doctors only when complications arise. Midwives must master fetal heart auscultation and other antenatal assessment techniques. Neonatal resuscitation is a critical competency within the midwifery scope, particularly for ensuring safety in emergency settings or resource-constrained environments like shelters. Midwives use SBE and low-fidelity mannequins to gain the physical dexterity required for newborn resuscitation and other life-saving interventions [11,13]. 

Midwives provide continuity of care that extends through the postpartum period, following the mother and newborn to monitor recovery and health. Their activities include routine gynecological screenings, breastfeeding support, and identifying potential post-birth complications [14]. Family planning is a key priority for ongoing educational reform. It is the integration of SRHR as a core midwifery skill. This enables midwives to take an independent lead in family planning and contraception, comprehensive sexuality education, and the prevention and management of STIs and HIV [15].

Systemic issues: Curriculum gaps feature limited SRHR breadth, hands-on training, and resources, and an outdated medical-model emphasis. Staff shortages, low pay, burnout, and war-related disruptions affect educators and students. Implementation of reforms is uneven.

Aligning With ICM/EU Standards While Addressing National Contexts and Wartime Realities

A practical approach to clinical skills teaching in midwifery emphasizes competency-based, student-centered methods that bridge theory and real-world practice. This integrates simulation, supervised clinical experience, structured feedback, and progressive skill-building, aligned with ICM Global Standards for Midwifery Education and Essential Competencies. The advances of online education revealed during the pandemic or wartime periods are documented by Akmese et al. [23].

Adopt a competency-based framework: Teaching should be based on ICM essential competencies for midwifery practice (e.g., antenatal care, labor support, normal birth, emergencies, postpartum/newborn care, and family planning). Clear, measurable learning outcomes need to be defined using frameworks like Bloom’s taxonomy (e.g., from imitation to mastery of skills like fetal heart auscultation or perineal repair). Around ~50% of the program should involve practical/clinical experience with sufficient time for integration, critical thinking, and continuity of care.

The use of structured skills and teaching models will enable effective step-by-step approaches [24,25]. Peyton’s four-step approach demonstrates (instructor shows), deconstructs (explains steps), enables comprehension (learner explains), and performance (learner practices under supervision). George’s five-step or Sawyer’s six-step frameworks ensure similar graduated responsibility, i.e., 'see one';' practice in controlled settings, prove competency, perform independently, and maintain skills. These frameworks, combined with instructional videos and live demonstrations for basic skills, will help improve performance and student satisfaction.

Simulation is a cornerstone for safe, repeatable practice, especially for rare emergencies or in resource-constrained settings (e.g., wartime Ukraine) [26]. Low-fidelity simulations involve part-task trainers and mannequins for skills like vaginal exams, suturing, or newborn resuscitation. High-fidelity simulation includes scenarios with birthing simulators, standardized patients, or role-play for teamwork, communication, and decision-making. Augmented/VR are emerging as tools for immersive practice. They help reduce anxiety, build confidence/dexterity, improve teamwork, and prepare for real emergencies. Use debriefing (e.g., structured reflection) after each session.

Follow Best Practices From the International Nursing Association for Clinical Simulation and Learning (INACSL) or ICM/Laerdal SimBegin Programs

The OSCEs need to be used for reliable assessment of discrete skills (e.g., stations for antenatal assessment, labor management, and emergency response) [27]. Task-based stations, decision-making scenarios, and values/evidence-based practice need inclusion. These need to be combined with portfolios, case studies, and direct observation for holistic evaluation.

Supervised clinical placements and preceptorship provide continuity of care experiences (following women through pregnancy, birth, and postpartum) rather than fragmented rotations. Preceptors/mentors need to be trained in teaching strategies, feedback, and supervision using models like Clinical Teaching Associate (CTA) for enhanced skill development and satisfaction. Targeted questioning, role modeling, deliberate practice, and immediate constructive feedback help strategise in clinical settings.

Additional practical strategies [28,29] include blended learning, i.e., lectures/demonstrations followed by simulation, followed by real practice. Peer-to-peer and student-led practice increases repetition using ICM competencies. Interprofessional education involves collaborating with obstetricians and nurses for realistic team scenarios. Feedback and reflection should be regular, specific feedback with reflective journals or debriefs. Resources should include adequate mannequins, simulation labs, updated equipment, and digital tools.

Prospects and Recommendations

Ukraine's midwifery education shift is part of a larger European convergence toward competent, autonomous, woman-centered practice, accelerated by EU integration goals. Progress includes targeted partnerships and higher education alignment, but full realization requires sustained investment in degree-level programs, curriculum overhaul per ICM standards, faculty development, and clinical capacity.

Key steps forward (drawing from UNFPA/ICM guidance) require the adoption of competency-based curricula with stronger SRHR integration; the strengthening of regulatory and professional associations; investment in educators, simulation/resources, and quality assurance; and leveraging of international collaborations for knowledge exchange. There is a well-established and growing space for simulation techniques in midwifery education. Simulation-based education is widely recognized as an effective, safe, and evidence-supported method to complement clinical placements, particularly for building clinical skills, confidence, and teamwork while protecting patients.

Evidence Supporting Simulation Training for Midwifery

Numerous systematic reviews and empirical studies emphasize that SBE provides a wide array of measurable advantages for midwifery training. One primary benefit is the significant improvement in technical competence and skill acquisition, as the controlled environment allowed by simulation enables students to develop dexterity in essential procedural tasks such as vaginal examinations, suturing, and newborn resuscitation.

Beyond manual skills, this pedagogical approach is crucial for cultivating non-technical competencies, including effective communication, situational awareness, and leadership, all of which are vital in high-stakes clinical environments where rapid decision-making and teamwork are required. Furthermore, simulation plays a vital role in the psychological preparation of students by reducing anxiety and building self-confidence before they enter real-world clinical settings.

From a safety perspective, simulation allows midwives to practice managing rare or highly complex obstetric emergencies without any physical risk to the mother or the infant, a benefit that has led to endorsements from global health bodies like the WHO. Ultimately, these methods lead to superior learning outcomes by boosting student motivation and performance, with the long-term educational and clinical impact being confirmed by a series of reviews spanning from the 2012 systematic review by Cooper et al. through to modern assessments by Elendu et al. in 2024 [30, 31].

Online education is not treated as a standalone solution but rather as a component of a 'blended learning' approach that must be integrated with intensive hands-on clinical experience. This is a practical strategy that combines theoretical instruction (such as lectures or demonstrations) with subsequent simulation and real-world practice. The use of instructional videos combined with live demonstrations for basic skills has been found to improve both student performance and satisfaction. Emerging digital tools like VR and augmented reality provide realistic, repeatable, and immersive practice environments that can boost student engagement and performance. These tools are particularly useful for practicing decision-making and teamwork in high-stakes scenarios. While digital tools are valuable, the sources emphasize that midwifery education is fundamentally competency-based and requires that approximately 50% of the program involve practical or clinical experience. This hands-on time is essential for bridging the gap between theory and real-world practice. In the context of wartime Ukraine, there is an accelerated need for adaptable training. Technology and simulation are seen as practical pathways to maintain higher-quality education despite the disruptions caused by the conflict.

While online or digital components like videos and VR are efficient for delivering theory and preparing students for clinical tasks, they cannot replace the 50% clinical placement requirement or the necessity of SBE for developing physical dexterity and safe practice. International organizations strongly advocate for it. The ICM integrates simulation into competency-based education frameworks and partners with Laerdal and others for skills labs aligned with ICM essential competencies. The WHO and UNFPA highlight SBE as a key strategy for strengthening midwifery education globally, including in resource-limited or conflict-affected settings. Simulation is not just 'a place' in midwifery education; it is a recommended, evidence-based component that enhances traditional training, especially for competency development and emergency preparedness. It is actively promoted by the ICM, WHO, and UNFPA and continues to evolve with technology. For programs in transition (like those in Ukraine), it offers a practical pathway to higher-quality, safer education.

Prospects and Future Directions

The outlook for Ukrainian midwifery is increasingly focused on academic advancement and professional autonomy. The aspiration for EU accession is a primary driver for updating professional qualification standards to meet EU Directive 2005/36/EC. The goal is to move away from a physician-led medical model toward woman-centered, physiological birth models that grant midwives greater independence. The prospect for midwives in Ukraine to become autonomous, responsible birth attendants is currently in a significant transitional phase, moving away from a historically physician-led medical model toward an independent, woman-centered professional standard.

Technological and Practical Support

The transition is being supported by SBE, which allows midwives to build the dexterity, decision-making, and situational awareness necessary for independent practice. This is particularly vital in the current context of war, where midwives must often act as the sole responsible attendants in emergency settings or shelters. The current trajectory of midwifery in Ukraine indicates a strong push toward enabling midwives to function as autonomous professionals in routine obstetrical care, gynecological care, and family planning, though this transition is still in progress.

Involvement in Routine and Specialized Care

The prospect for midwives to act as autonomous persons extends across several areas of care. In obstetrical care, the focus is shifting toward woman-centered, physiological birth. In this model, midwives independently manage normal pregnancies and deliveries, referring to medical doctors only when complications arise. Family planning and gynecological care. Competency-based education reforms, supported by the WHO and UNFPA, are designed to include SRHR as core midwifery skills. This includes independent involvement in family planning and contraception, routine gynecological screenings and care, and prevention and management of STIs/HIV. While midwives have traditionally been dependent on doctors in a medicalized hierarchy, the clear goal of ongoing reforms and international partnerships is to establish them as independent, responsible attendants for routine maternal and reproductive health services.

## Conclusions

Midwifery in Ukraine is currently in a significant transitional phase, moving away from its historical dependency on a medicalized hierarchy toward a future of professional autonomy and academic advancement. The path forward is defined by the elevation of the profession to the BSc, MSc, and PhD levels, which is essential for building research capacity, leadership, and evidence-based clinical practice. While substantial challenges remain, including acute workforce shortages, curriculum gaps, and the ongoing impact of conflict, the clear trajectory is toward full integration into the EHEA and alignment with EU directive 2005/36/EC. To achieve this, sustained investment is required in competency-based education, faculty development, and the strengthening of professional bodies like the Ukrainian Midwives' Union. Ultimately, establishing midwives as independent, responsible birth attendants is not only a matter of professional status but also a critical strategy for improving maternal and newborn health outcomes across Ukraine.
